# Genomic epidemiology of clinical ESBL-producing *Enterobacteriaceae* in a German hospital suggests infections are primarily community- and regionally-acquired

**DOI:** 10.1099/mgen.0.000901

**Published:** 2022-12-16

**Authors:** Lisa Neffe, Taya L. Forde, Katarina Oravcova, Ute Köhler, Wilfried Bautsch, Jürgen Tomasch, Susanne Häussler

**Affiliations:** ^1^​ Department of Molecular Bacteriology, Helmholtz Center for Infection Research, Braunschweig, Germany; ^2^​ Institute for Molecular Bacteriology, TWINCORE GmbH, Center of Clinical and Experimental Infection Research, a joint venture of the Hannover Medical School and the Helmholtz Center for Infection Research, Hannover, Germany; ^3^​ Institute of Biodiversity, Animal Health and Comparative Medicine, University of Glasgow, Glasgow, UK; ^4^​ Städtisches Klinikum Braunschweig gGmbH, Germany; ^5^​ Department of Clinical Microbiology, Copenhagen University Hospital – Rigshospitalet, Copenhagen, Denmark; ^6^​ Cluster of Excellence RESIST (EXC 2155), Hannover Medical School, Hannover, Germany

**Keywords:** epidemiology, *E. coli*, ESBL, hospital, *K. pneumoniae*

## Abstract

Clinical *Enterobacteriaceae* isolates that produce extended-spectrum β-lactamases (ESBLs) have been increasingly reported at a global scale. However, comprehensive data on the molecular epidemiology of ESBL-producing strains are limited and few studies have been conducted in non-outbreak situations.

We used whole-genome sequencing to describe the population structure of 294 ESBL-producing *Escherichia coli* and *Klebsiella pneumoniae* isolates that were recovered from a German community hospital throughout a 1 year sampling period in a non-outbreak situation.

We found a high proportion of *E. coli* isolates (61.5 %) belonged to the globally disseminated extraintestinal pathogenic ST131, whereas a wider diversity of STs was observed among *K. pneumoniae* isolates. The *E. coli* ST131 population in this study was shaped by multiple introductions of strains as demonstrated by contextual genomic analysis including ST131 strains from other geographical sources. While no recent common ancestor of the isolates of the current study and other international isolates was found, our clinical isolates clustered with those previously recovered in the region. Furthermore, we found that the isolation of ESBL-producing clinical strains in hospitalized patients could only rarely be associated with likely patient-to-patient transmission, indicating primarily a community and regional acquisition of strains.

Further genomic analyses of clinical, carriage and environmental isolates is needed to uncover hidden transmissions and thus discover the most common sources of ESBL-producing pathogen infections in our hospitals.

## Data Summary

Raw sequence files have been deposited to NCBI under accession number PRJNA769856 (http://www.ncbi.nlm.nih.gov/bioproject/769856). Strains are available from the authors upon request. Supplementary Material can also be found in the figshare account https://figshare.com/s/3cedd3139a18135cad88.

**Impact Statement**

Rising numbers of ESBLs have been reported over the last years from *Enterobacteriaceae* in hospitals. We generated comprehensive data on the molecular epidemiology of ESBL-producing strains. Our results primarily indicate a community and regional acquisition of ESBL strains in hospitalized patients. This study contributes towards future infection control measures.

## Introduction

Cephalosporins are beta-lactam antibiotics used to manage a wide range of infections. However, since their first description in 1983, clinical isolates that produce extended-spectrum β-lactamases (ESBLs) have been increasingly reported [1, 2]. This rise appears to be largely due to ESBL producing *Escherichia coli* (ESBL-Ec) and *Klebsiella pneumoniae* (ESBL-Kp) [2, 3]. For example, in Germany, the proportion of ESBL-Ec isolates increased from 4.8 % in 2008–12 % in 2019 [4]. ESBL-producing strains commonly express CTX-M enzymes, which hydrolyse penicillin, narrow-spectrum and third-generation cephalosporins, as well as monobactams [2].

Two important shifts in the ESBL-Ec epidemiology have been recognized in hospitals worldwide in the past years. The *E. coli* sequence type (ST) 131 sublineage clade C (sub-clade C2/H30Rx), which harbours *bla*_CTX-M-15_ *,* emerged in the 1990s. Since then it spread rapidly throughout the world [5–7] and became globally dominant [8]. Today a second subclade (C1/H30R) carrying *bla*_CTX-M-27_ *,* is increasingly detected and clonally spread across Europe, including Germany [9–11]. In addition, fluoroquinolone resistant emerging ESBL-Ec of ST1193, are described in several parts of the world since 2012 [12]. In Germany this emerging ST was identified for the first time in 2015 [13].

Comprehensive global data on the molecular epidemiology of ESBL-producing *Enterobacteriaceae* are currently limited and only a few studies have been conducted in non-outbreak situations. Here, we used whole-genome sequencing (WGS) to describe the molecular characteristics and phylogenetic relatedness of 234 ESBL-Ec and 60 ESBL-Kp isolates, which were obtained from a German community hospital throughout a 1 year sampling period in a non-outbreak situation. Our extensive WGS data allowed us to describe the overall genomic population structure and enhanced our understanding of the transmission dynamics of these problematic nosocomial pathogens.

## Methods

### Clinical isolate and epidemiological data collection

All ESBL-Ec and ESBL-Kp isolates that were recovered between April 2019 and April 2020 at the diagnostic microbiology lab of a large German community hospital (approximately 1500 beds, distributed over 57 wards in the central hospital and 13 wards outside the campus) were included in this study (Table S2A, C). Phenotypic resistance testing and interpretation was done based on the EUCAST guidelines implemented in the VITEK2 system v8.02 (parameter set 2018 and 2019). All ESBLs were streaked on a 5 % Columbia sheep blood agar plate for transfer. Sixty of more than 100 *K *. *pneumoniae* isolates and 234 of approximately 300 *E. coli* isolates could be re-cultured from the transfer plates and were subjected to WGS using an Illumina short read sequencing approach.

The date of isolate recovery from patients’ samples was recorded, as well as information on the dates of the patients’ admission and discharge, the hospital ward, and the patients’ room at the time of sampling. Records of previous ESBL carriage were considered for the evaluation of nosocomial versus community acquired infections. Nosocomial infections were defined as those diagnosed after 48 h of hospital admission.

### Whole-genome sequencing, assembly and bioinformatics analyses

Short-read WGS library preparation of the DNA of all clinical isolates was based on a modified version of the Illumina Nextera XT protocol [14, 15]. We conducted 150 bp paired-end sequencing on the NextSeq500 or NovaSeq6000 instrument. Low quality (Phred score <30) and adapter contaminations were removed using Trimmomatic v0.32 [16]. Paired and unpaired reads were assembled with Spades v3.13.0 [17] in default mode. Sequencing statistics for ESBL-Ec and ESBL-Kp strains are provided in Tables S2B and D, respectively.

For long-read sequencing on the MinION device (Oxford Nanopore Technologies), performed for five ESBL-Ec and three ESBL-Kp (Table S1, available in the online version of this article), high molecular weight genomic DNA was extracted from 1.5 ml overnight culture grown in LB using the phenol/chloroform method [18]. Libraries were prepared with the SQK-LSK109 and EXP-NBD104/114 barcoding kits (both Oxford Nanopore Technologies) according to the manufacturer’s instructions. *Guppy* v3.1.5 (ONT) was used for high accuracy base calling. *Unicycler* v0.4.8 [19] was used with default settings to reconstruct closed genomes from short- and long-read sequences.

*In silico* multi-locus sequence typing (MLST) was performed, based on the ‘Achtman scheme’ using *pubMLST* v2.19.0. Details on annotation, pangenome analysis, allele typing and antimicrobial resistance gene detection are provided in Supplementary Text S1.

### Phylogenetic analysis

An initial core genome alignment and phylogenetic tree of all isolates of a species was generated with *parSNP* [20]. We extended the core genome alignment approach and focused on comparative analysis of closely related isolates. In order to increase the proportion of core positions and thus enhance the phylogenetic resolution [21–23], sequence reads of isolates were mapped to a closed reference genome of the respective ST using snippy v4.6.0 [24] as detailed in Suppl. Text S1. High quality closed genomes of the major STs served as a reference for short sequence read mapping of the isolates of the respective ST. When no associated genome was publicly available for an ST, we performed long-read sequencing of a representative of that ST from our collection of strains to obtain closely related reference genomes of high quality (listed in Table S1). Overall, seven *E. coli* STs and four *K. pneumoniae* STs were included in the analysis, which amounted to 203 ESBL-Ec isolates and 32 ESBL Kp isolates, respectively. Maximum likelihood trees were generated with RAxML based on general time-reversible model of substitution and a gamma distribution to account for rate heterogeneity between sites. Trees were visualized and annotated in iTOL [25].

## Results

### Population structure of ESBL-producing *Enterobacteriaceae* isolated from a hospital setting

In total, 234 ESBL-Ec isolates from 179 patients were included in this study. One hundred and thirty-eight isolates came from patients that were only sampled once, while 41 patients were sampled multiple times, ranging from two to four times. The majority (67 %) of the ESBL-Ec isolates were isolated from urinary tract samples, 9 % of isolates were recovered from wounds or intraoperative sites, 8 % from the nasopharynx, 6 % from blood stream infections, and 9 % isolates from other sites. Three isolates (1 %) were isolated from rectal swabs (non-infecting isolates). In 24 % of all patients, the pathogen it was isolated 48 h after admission to the hospital, and the infection was therefore categorized as nosocomial. Half of the patients were classified as already infected upon admission with ESBL-Ec. In total, 26 % of patients were treated in wards outside the central campus of the hospital. For those patients no information on previous carriage or infections with ESBL producing isolates was available (Table S2A).

**Fig. 1.**
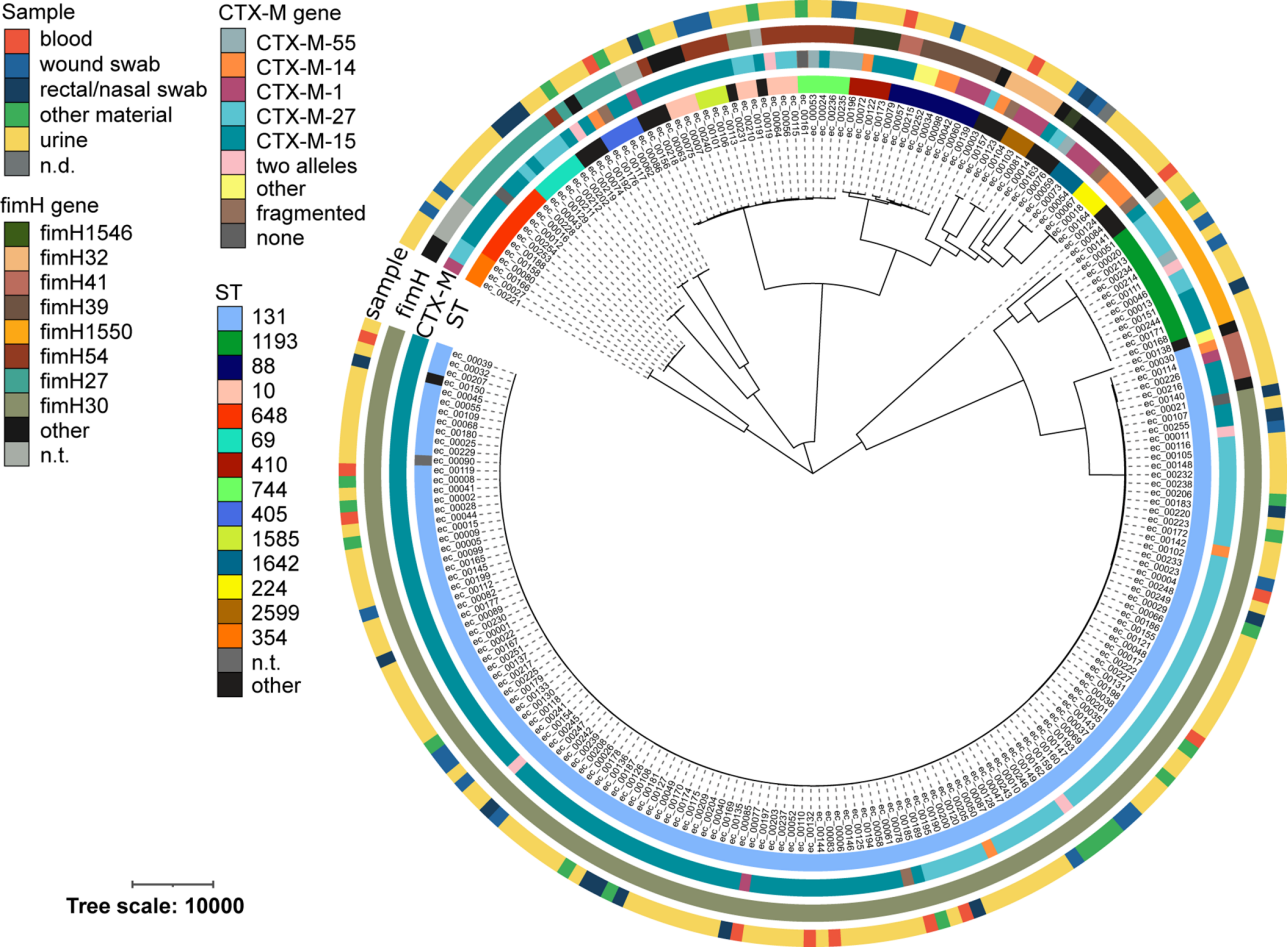
Core genome phylogeny and genomic features of clinical ESBL-Ec isolates. Maximum likelihood phylogenetic tree (unrooted) of 234 *E. coli* genomes based on a 2 451 162 bp core alignment generated with parSNP. Isolates (branch tips) are colour-coded according to their sequence-type (ST), the presence of different *_bla_CTX-M* and *fimH* alleles, and the origin of the sampled material, as indicated in the figure key (left, from inner to outer ring). The ST was based on multi-locus sequence typing (MLST) annotation using pubMLST. The clonal complex of ST10 comprises isolates of different STs. In five isolates we detected only parts of *_bla_CTX-M* genes (below 90 % coverage), which could be due to fragmented assemblies. n.t. – not typeable, n.d. – no data available.

The evolutionary relationships among the 234 clinical ESBL-Ec were inferred through a *de novo* assembly-based core genome alignment and subsequent maximum likelihood (ML) tree (Fig. 1). Strains consistently clustered according to their MLST, with the exception of ST10, ST1585 and ST744, which formed a single monophyletic cluster. In total 31 different STs were found. The majority of the ESBL-Ec belonged to ST131 (*n*=144, 61.5 %), which was strongly associated with the presence of the *fimH30* allele. The second most prevalent ST (*n*=12) was the emerging ST1193 harbouring the *fim*H1550 allele. The ESBL phenotype was associated in the majority of ESBL-Ec (231/234) with the presence of a *bla_CTX-M_* gene; *bla_CTX-M-_*_15_ was detected in 51 % and *bla_CTX-M-_*_27_ in 29 % isolates.

The 60 ESBL-Kp strains were isolated from 49 patients overall. Fourty-one patients were sampled only once, while 19 isolates were obtained from patients, who were sampled multiple times. Eight patients (16 %) developed an infection with ESBL-Kp 48 h after admission to the hospital (nosocomial), 23 (46 %) carried the isolate upon admission or had a record of permanent ESBL carriage. For 19 patients (38 %) this information was not available. More than half of the ESBL-Kp isolates were isolated from urinary tract samples (55 %), 8 % from blood, one isolate from the nasopharynx and 35 % from other sites (Table S2C). The majority of the 60 *K *. *pneumoniae* isolates (80 %) belonged to ten dominant STs, including the globally prevalent ST1626, ST14, ST307 and ST48 (Fig. S1).

### ST-specific accessory genome content in ESBL-producing isolates

For the ESBL-Ec isolates included in this study, the pan-genome contained in total 15 414 genes, 12 542 of which (81%) belonged to the accessory genome. The pan-genome of the 60 ESBL-Kp isolates contained 10 065 genes, 60 % of which (6084 genes) were accessory. The gene accumulation curves of the ESBL-Ec and ESBL-Kp pan-genomes depict open pan-genomes for both species (Fig. S2). Nevertheless, the ESBL-Ec accumulation slope appears to be levelling off, indicating that with the 234 sequenced isolates taken into account, fewer new *E. coli* genes would be discovered by sequencing additional isolates.

**Fig. 2.**
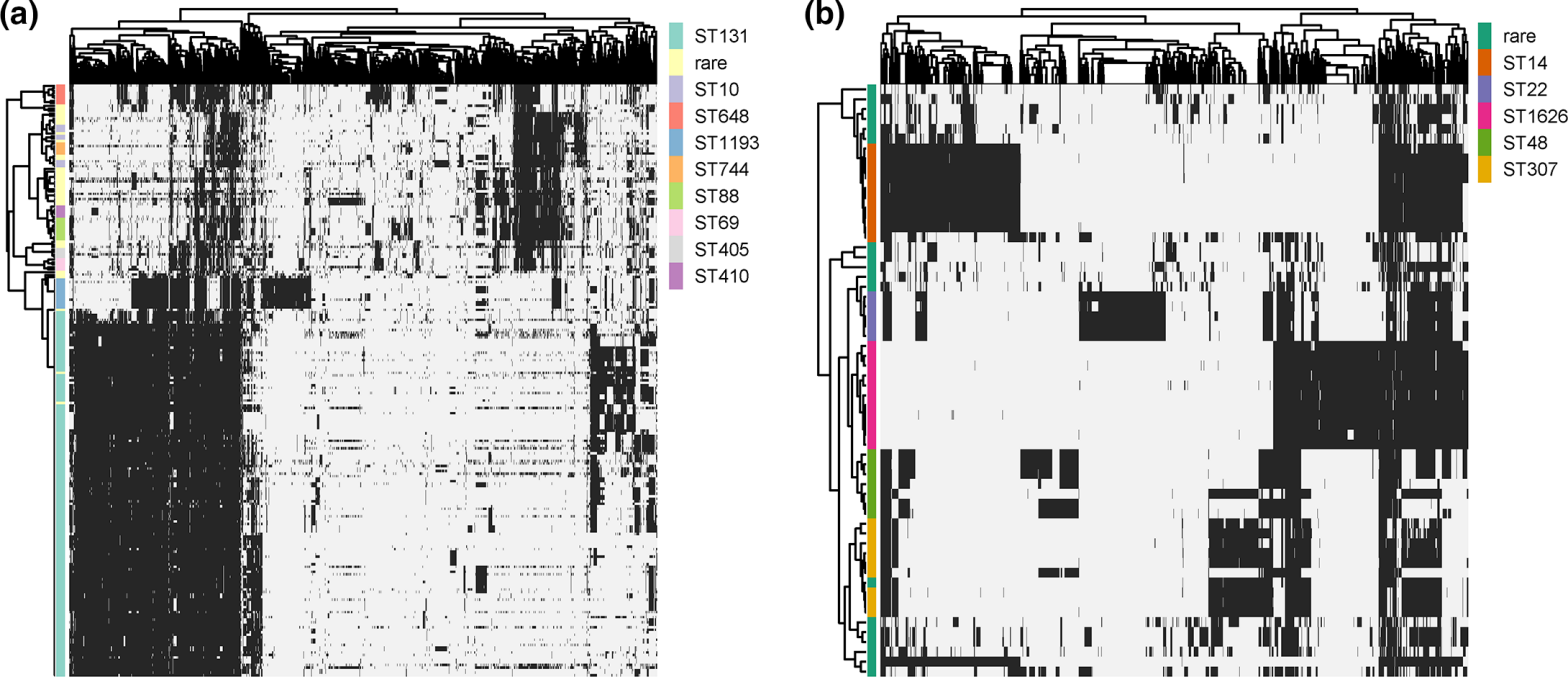
Presence/absence matrix of accessory genes of ESBL*-*producing clinical isolates. (**a**) Clustering of the presence/absence of 4704 common ***E.** coli* accessory genes (present in 5–95 % of the 234 clinical *E. coli* isolates analysed in this study). (**b**) Clustering of the presence/absence of 2506 common *K. pneumoniae* accessory genes (present in 5–95 % of the 60 clinical *K. pneumoniae* isolates analysed in this study). On the left: ML tree, based on the core genome alignment. The clinical isolates are colour-coded according to their sequence-type as indicated in the figure key, rare: combined data of rare STs with fewer than three members.

The overall presence/absence pattern of the accessory genes of both *E. coli* and *K. pneumoniae* largely reflected the affiliation to a particular ST (Fig. 2). This indicates that phylogenetically related isolates tend to have similar accessory genome content. In line with this finding, the pairwise gene content differences (Jaccard distances) between the clinical isolates and the pairwise SNP distances in their core genes were significantly correlated. While the correlation coefficient was 0.92 (*P*-value=0.001, Mantel test) for the ESBL-Ec isolates, it was slightly lower for the ESBL-Kp isolates (correlation coefficient=0.81, *P*-value=0.001).

### Genetic relatedness of ESBL-Ec isolated from the same versus different patients

Ninety-six of the 234 ESBL-Ec isolates were derived from multiple samplings from single patients. ESBL-Ec isolates of up to three different STs were recovered from a single patient (patient AA [arbitrary identification letters],Table S2). A further three patients (AC, AD, BN) harboured ESBL-Ec isolates that belonged to two different STs. In all other patients (*n*=35), at least two ESBL-Ec isolates from the same ST were recovered. Fig. S3 depicts the SNP differences between ESBL-Ec isolate pairs within all major ST. While most of the isolates from the same patient exhibited SNP differences below ten, there were two exceptions. In one patient (AR), from whom three isolates were recovered within a maximum time span of 10 months, we observed SNP differences of up to 57, and in the second patient (AP) the SNP difference was 15 in two isolates obtained on two consecutive days from different body sites (nasopharyngeal swab and ascites).

**Fig. 3.**
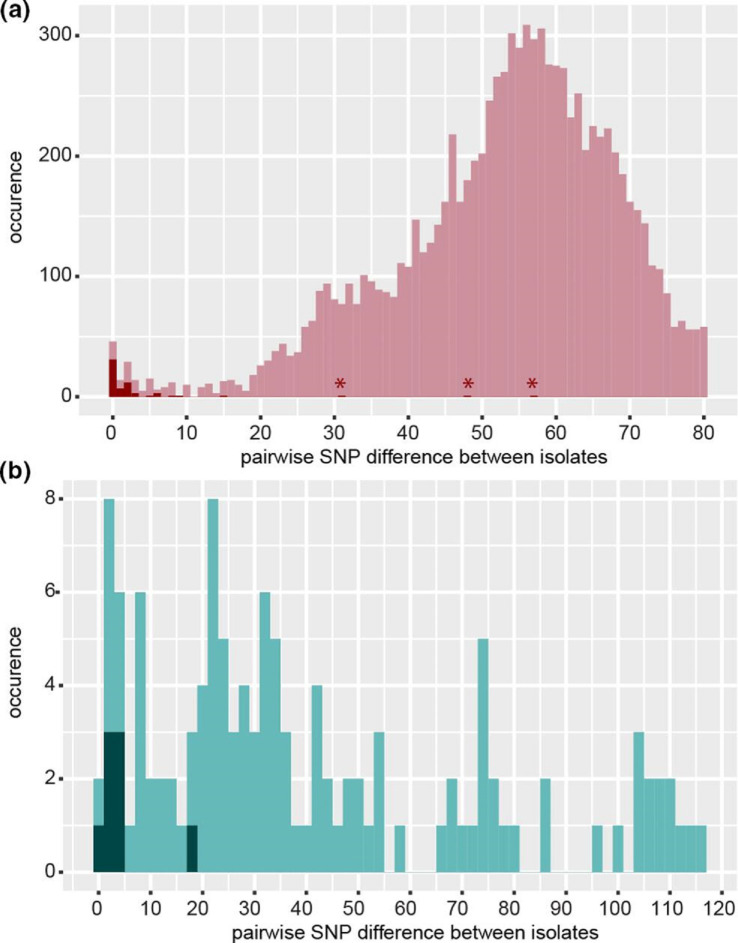
Distribution of the pairwise SNP differences between all ESBL-Ec and ESBL-Kp isolates of the major STs. (**a**) The pairwise SNP differences between 203 *E. coli* isolates of ST1193, ST88, ST410, ST648, ST69 and ST10 clonal complex were calculated and plotted. Many of the isolates were recovered from different body sites, some of them as part of screening. Dark red: SNP differences of pairs of isolates from patients that were sampled repeatedly. The median sampling interval was 9 days with a maximum of 10 months between the first and the last isolate. Asterisks highlight isolate pairs from the same patient (AR), which exhibited the highest SNP distances. (**b**) The pairwise SNP differences between 32 ESBL-Kp isolates from the same ST type (ST1626, ST22, ST48 and ST307) were calculated and plotted. Dark green: SNP difference of pairs of isolates from six patients that were sampled repeatedly. The median sampling interval was 22 days with a maximum of 5 months between the first and the last sampling. The SNP differences between the overall 14 isolates of these patients were all below six SNPs, with the exception of one ESBL-Kp isolate pair with a SNP difference of 19 (patient AJ, sampled within 3 days). Isolates of the major STs of ESBL-Kp revealed no clear local minimum around a SNP difference of ten.

We next calculated the distribution of the pairwise SNP differences between ESBL-Ec and Kp isolates of all major STs (Fig. 3). We observed increasing numbers of isolates that differed by more than 20 SNPs when recovered from different patients.

### Use of epidemiological and genomic data to investigate the likelihood of patient-to-patient transmission

**Fig. 4.**
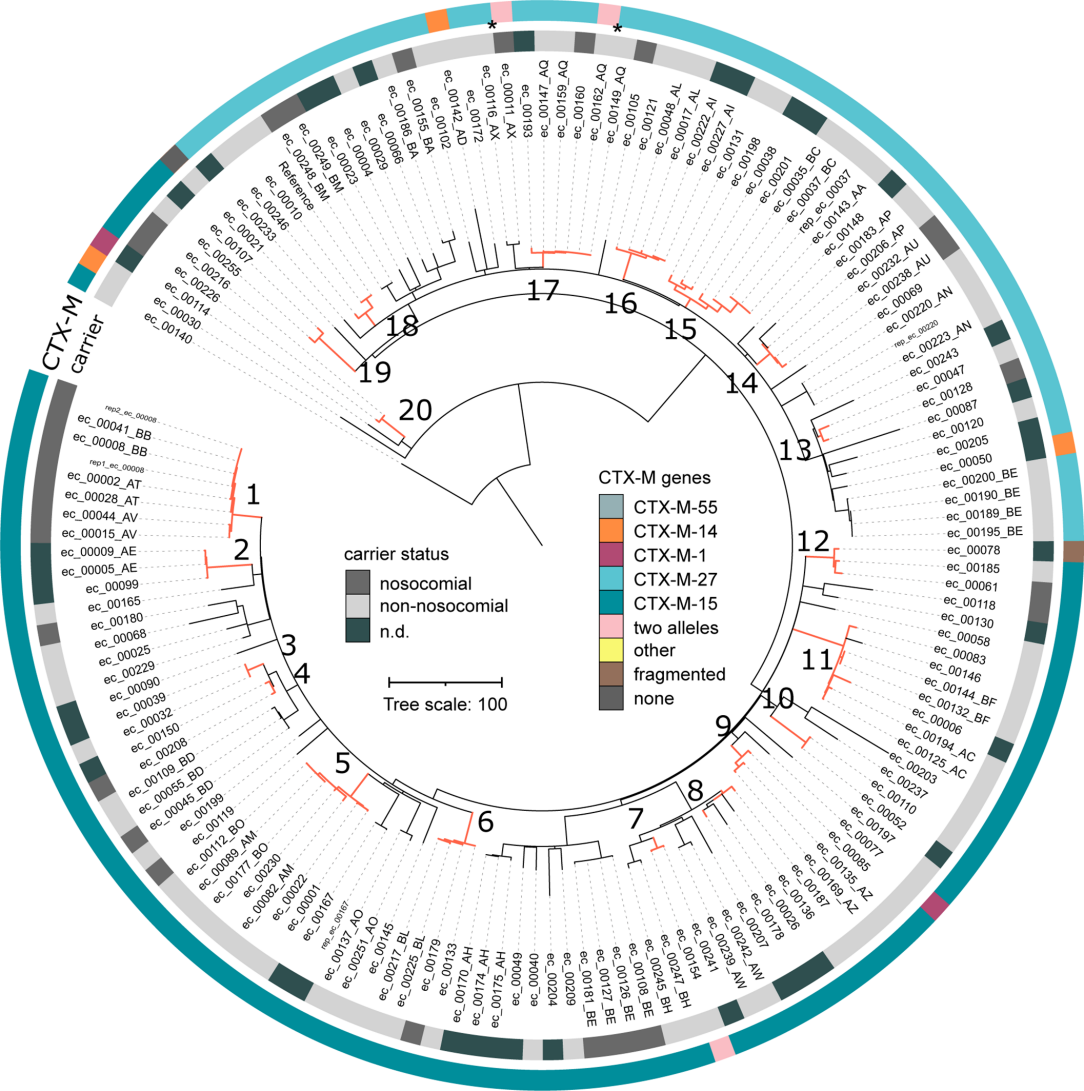
Core genome phylogeny and genomic features of ESBL-Ec ST131 isolates. Maximum likelihood phylogeny of *E. coli* ST131 genomes built with RAxML 8.2.12. Tree was based on the recombination-free alignment of 3825 bp. The ec_0140 isolate (ancestral clade B) was used as an outgroup. The clade A branch has been shortened. Isolates (branch tips) are colour-coded according to the presence of different *_bla_CTX-M* alleles and the status of carriage upon hospital admission, as indicated in the figure key from outer to inner ring. Arbitrary two-letter codes were assigned to individual patients so that isolates that were recovered from the same patients can be followed. Detection of fragmented *_bla_CTX-M* genes (below 90 % coverage) is indicated. Nosocomial: isolates that were recovered from patients >48 h after admission. Non-nosocomial: isolates that were recovered from patients within the first 48 h after admission. N.d. no data available. The asterisks highlight two isolate pairs that were recovered from the same patient and which were very closely related (0 and three SNP difference, respectively). However, in one patient, the later isolate harboured an additional *_bla_CTX-M-15* copy and in the other an additional *_bla_CTX-M-1* copy. Twenty sub-clusters (SCs) were identified (indicated by numbers). In those SCs the genomes of the respective isolates (red lines), differed by fewer than ten SNPs.

The ML tree of the 144 ST131 isolates (Fig. 4) shows 20 sub-clusters within which the isolates (overall 75) differed by fewer than ten SNPs. While many of those isolates were recovered from the same patients, all 20 sub-clusters also contained isolates from different patients (Fig. S3). Interestingly, we found two isolate pairs that – although very closely related (0 and three SNP differences, respectively) – acquired different *CTX-M* genes; in one patient the later isolate harboured an additional *bla_CTX-M-_*_15_ copy (patient AQ) and in the other an additional *bla_CTX-M-1_* copy (patient AX).

Investigation of the other ESBL-Ec STs (ST1193, ST88, ST410, ST69, clonal complex ST10) also revealed sub-clusters of isolates with fewer than ten SNP pairwise distances recovered from different patients (Fig. S4). Likewise, among the 32 strains of ESBL-Kp ST1626, ST22, ST48 and ST307 we identified four sub-clusters containing 12 isolates from different patients with a SNP difference of less than ten (Fig. S5).

The ST131 sub-cluster 1 (SC-1) comprised six strains that were recovered from three patients. Remarkably, those three patients were treated on the same intensive care unit (ICU), they were hospitalized at the same time, and all strains were recovered within a short time period (1 month). The six strains differed by a maximum of three SNPs (Fig. S6), indicating that direct patient-to-patient transmission could have taken place. Furthermore, we found three ST1193 isolates that differed by only four SNPs and that were obtained from two patients who were treated on the same ICU at the same time (Fig. S4). We also found two ST69 isolates, exhibiting only two SNP differences that were recovered from a mother and her premature infant. In a fourth case we found two identical ST10 clonal complex isolates (0 SNP difference) sampled on consecutive days from one stationary and one outpatient. While it is unlikely that the patients were in direct contact, both isolates were from swabs taken during surgery, thus a common environmental source cannot be excluded.

We could identify only one possible epidemiological link between two patients with closely related ESBL-Kp isolates (ST22). The two patients stayed in the same room for 6 days (eight SNPs difference between their isolates). A similar isolate was found in a third patient who was hospitalized at the same time as one of the two other patients, but on a different ward (two SNPs difference between their isolates).

We could not identify an epidemiological link for any of the remaining isolates within the Ec or Kp sub-clusters, despite the fact that the strains were very closely related (less than ten SNP differences). The patients from whom the isolates were recovered were not hospitalized at the same time, and they were not treated in the same ward. Interestingly, the median number of days between the isolation of any of two strains belonging to one of the 20 ST131 sub-clusters and those of the strains outside of the sub-cluster were not significantly different (104 versus 110 days, Wilcoxon rank sum test, *P*-value=0.34). We also did not identify a significant difference in the time interval between the isolation of closely-related versus more distantly related strains among the other (non-ST131) ESBL-Ec STs (98 versus 110 days, Wilcoxon rank sum test, *P*-value=0.09), or the ESBL-Kp STs (102 versus 104 days). This lack of an overall temporal association in the isolation of strains within the sub-clusters argues against frequent direct patient-to-patient transmission in the hospital.

### Study isolates in the context of national and international ESBL-Ec isolates

We generated a phylogenetic tree of the *E. coli* isolates of our study and 694 publicly available ST131 clade C strains. In this analysis we included all genome sequences of Eurasian ST131 isolates from the EnteroBase [26] that were published since 2001, and which fulfilled our quality criteria, and that covered at least 75 % of the reference genome ec_00010. Thirty-seven of those isolates were clinical ST131 isolates from Germany, recovered between 2009 and 2016 [11, 27].

**Fig. 5.**
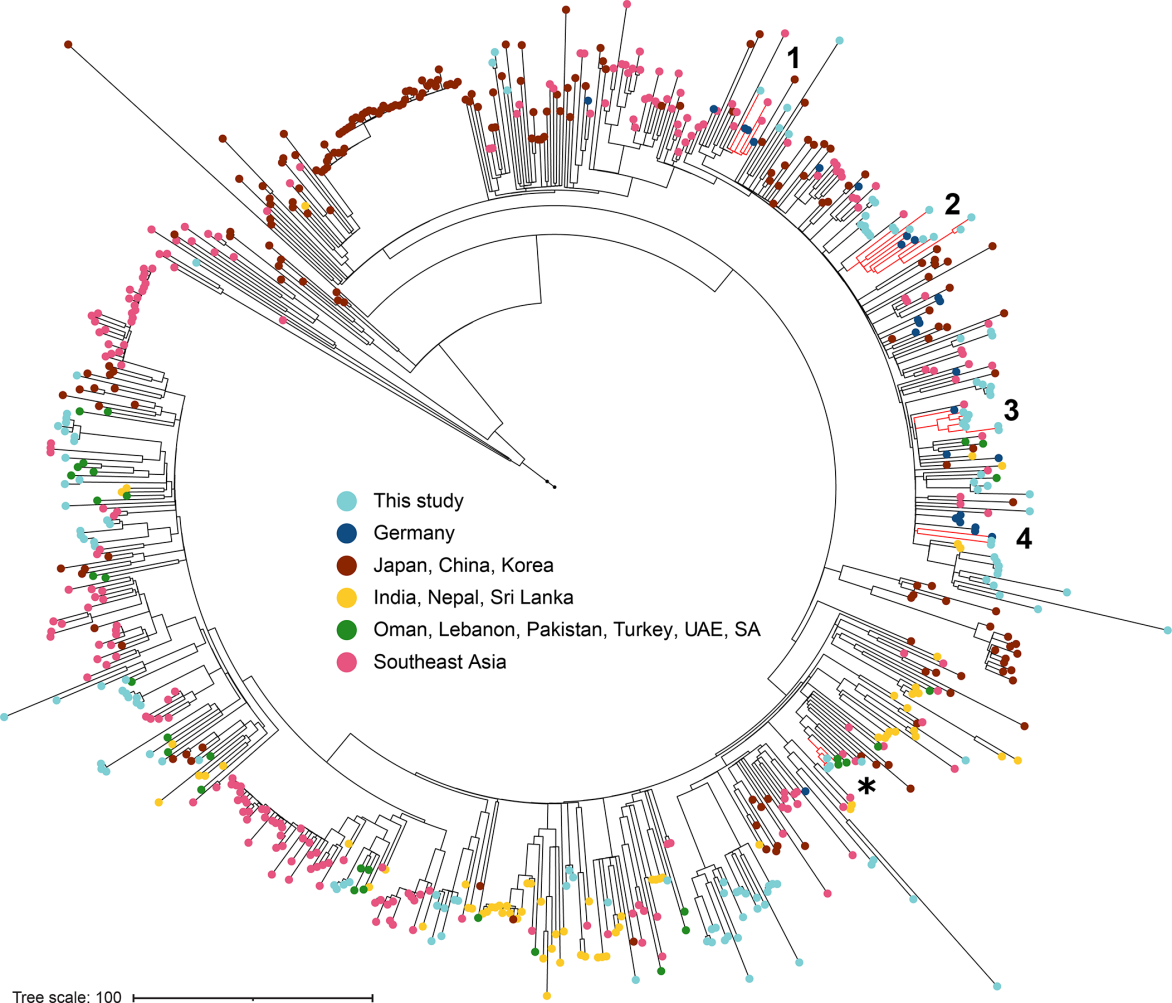
Core genome phylogeny of a global collection of ST131 clade C strains. Maximum likelihood phylogeny of *E. coli* ST131 genomes built with RAxML 8.2.12. This tree is based on a recombination-free alignment of 15 356 bp. It comprises 656 ST131 clade C Asian strains, 37 strains that were previously recovered in Germany [11, 26] as well as the ST131 clade C isolates of the current study. The branch tips are coloured according to the region in which the strain was isolated. Numbers indicate clades consisting of isolates of the present study as well as previously published from Germany. Asterisk indicates the smallest SNP difference between an international isolate and strains from the current study. UAE: United Arabic Emirates, SA: South Africa.

The isolates from this study formed several clusters, which were widely distributed across the broader ST131 phylogenetic tree (Fig. 5). However, none of the international isolates and isolates of the current study shared recent common ancestors. This indicates that ongoing multiple and direct importations from various geographic regions does not seem to be responsible for the cases observed in our hospital. Interestingly, several of the ST131 clusters of this study included isolates that were previously recovered in northern Germany [11], indicating transmission of strains throughout the wider region of northern Germany. SNP differences as small as 15 were observed between clinical ST131 strains of this study and previously published strains (ec_00105 and ERR1999747).

The smallest SNP distance between an international isolate and an isolate of the current study was 11 SNPs (between eco_bs_00085 and SRR7828835 from Turkey). Further highly similar clinical isolates were from 2015 and 2017, from Saudi Arabia (SRR7828743) and Singapore (SRR7828766).

## Discussion

This study aimed at describing the population structure of ESBL-Ec and ESBL-Kp recovered from a German community hospital in a non-outbreak situation. We found that more than 60 % of the ESBL-Ec isolates belonged to the human associated ST131, which became predominant in the early 2000s [5]. In 2016 a variant of the ST131 C lineage, the C1/H30R subclade, carrying *bla_CTX-M-_*_27_, was described [28]. Since then this variant has been increasingly found in Europe [10, 11]. In this study 29 % of the ST131 isolates belonged to this C1 sub-clade (Fig. 1). Further studies will have to elucidate whether individual genes or genomic regions within the accessory genome can be identified as major drivers of the success of this and other globally disseminating high-risk clones. In this context, it is interesting that in this study we found a high overall correlation of the core and accessory genome for both Ec and Kp ESBLs. There seems to be a ST-specific accessory genome in ESBL-producing isolates beyond the well-described association of certain lineages with distinct CTX-M alleles [21, 29, 30].

A second well-described observation is a global increase in clinical ST1193 isolates since 2012 [13]. ST1193 has been associated with fluoroquinolone resistance and was identified in Germany for the first time in 2015 [12, 13]. ST1193 accounted for 5 % of the ESBL-Ec isolates in our study. We detected four different *bla_CTX-M_* alleles in the ST1193 ESBL-Ec isolates. Allele *fim*H64 is a clonal marker associated with ST1193 [12, 13]. However, all our ST1193 isolates had an allele *fim*H1550 suggesting that the ST1193 genome is more diverse than previously thought.

In addition to reporting on the population structure of ESBL-producing isolates from this study, we applied a genomic epidemiology approach to identify potential patient-to-patient transmissions. Similar to other studies [31–34], suspected patient-to-patient transmissions of ESBL-producing isolates were rare. We identified only a few cases in which, in addition to a high similarity of the genomes of the isolated ESBL-producing strains (below ten SNP differences), a possible contact between patients was likely. It seems that the great majority of the patients acquired the ESBL producing strains outside of the hospital. Approximately half of the ESBL-isolates were recovered from the patients within 48 h of admission, indicating a community-acquired infection. This rate might be even higher, as for 26 % (ESBL-Ec) and 38 % (ESBL-Kp) of the isolates, information on the date of patient admission was not available and patients were not screened for ESBL-carriage upon admission.

Nevertheless, limitations of this study might also lead to an underestimation of the transmission rate. ESBL-Ec and ESBL-Kp isolates from patients that were sampled repeatedly generally showed a low SNP distance. However, some isolate pairs exceeded the similarity threshold of ten SNPs, a threshold which has also been applied in other studies [34], while the phylogeny in these cases clearly confirmed a recent common ancestor. In order to avoid underestimation of transmission events, absolute distance thresholds should be applied carefully, and information on the descent of isolates provided by the topology of the tree might have to be taken into account [35, 36]. In addition, we did not collect patient movement data, and conducted limited sampling, thus very likely underestimating patient contacts and transmissions [37–39]. Furthermore, asymptomatic carriers, and isolates from the hospital environment, staff and medical equipment as a potential source of clinical infections, were not included in the reconstruction of transmission chains.

The contextualization of our ESBL-Ec ST131 against 693 published ST131 genomes from various geographic origins implies that there were multiple introductions of different clonal linages of this globally spread pathogen into the region. However, the international isolates and isolates of the current study did not show recent common ancestors, suggesting that the cases observed in the hospital are unlikely to have resulted from recent imports. Interestingly, the finding that our strains were more closely related to clinical strains that had been previously isolated from different regions of Germany indicates that circulation of strains throughout the wider region is commonplace. Nursing homes and long-term care facilities could be a possible link in the dissemination of ESBL-Ec and ESBL-Kp between the hospital and the community settings [40, 41]. It is expected that contextual data from other sources can improve local genomic surveillance at multiple levels and uncover hidden transmission links in order to unravel the most common infection sources in patients with ESBL-producing *Enterobacteriaceae* in our hospitals.

## Supplementary Data

Supplementary material 1

Supplementary material 2
