# Supplementary material for: Genomic epidemiology of clinical ESBL-producing Enterobacteriaceae in a German hospital suggests infections are primarily community- and regionally-acquired
Source: Microb Genom. 2022 Dec 16;8(12):mgen000901. doi: 10.1099/mgen.0.000901 (PMC9837565; doi:10.1099/mgen.0.000901)
Supplement: Supplementary material 1 [file mgen-8-901-s001.pdf]

Supplementary Information

This file contains Supplementary Text S1, Legends for supplementary figures S1-S6, the legend for supplementary tables S1-S2

Genomic epidemiology of clinical ESBL-producing Enterobacteriaceae in a German hospital suggests infections are primarily community- and regionally-acquired

Lisa Neffe[1,2], Taya L. Forde[3], Katarina Oravcova[3], Ute Köhler[4], Wilfried Bautsch[4], Jürgen Tomasch[1], Susanne Häussler[1,2,5,6]*

[1]Department of Molecular Bacteriology, Helmholtz Center for Infection Research, Braunschweig, Germany.

[2]Institute for Molecular Bacteriology, TWINCORE GmbH, Center of Clinical and Experimental Infection Research, a joint venture of the Hannover Medical School and the Helmholtz Center for Infection Research, Hannover, Germany.

[3]Institute of Biodiversity, Animal Health and Comparative Medicine, University of Glasgow, Glasgow, United Kingdom.

[4]Städtisches Klinikum Braunschweig gGmbH, Germany.

[5]Department of Clinical Microbiology, Copenhagen University Hospital – Rigshospitalet, Copenhagen, Denmark

[6]Cluster of Excellence RESIST (EXC 2155), Hannover Medical School, Hannover, Germany

*correspondence Susanne.Haeussler@helmholtz-hzi.de

Supplementary Text S1 - Methods Phylogenetic analysis

Draft assemblies were analysed using QUAST v5.0.2 (Gurevich et al. 2013). To obtain an initial phylogenetic tree of all isolates of the species a core genome alignment, based on high quality assemblies, was generated with *parSNP* using default parameters using an internal reference (Treangen et al., 2014). *In silico* multilocus sequence typing (MLST) was performed, based on the “Achtman scheme” using *pubMLST* v2.19.0.

To subsequently generate a higher resolution phylogenetic tree of certain STs, the isolates were subjected to a reference based mapping approach using *snippy* v4.6.0 (Seemann, 2015). An initial alignment calculated by *snippy-core* was analyzed regarding false positive positions in the hybrid assemblies that served as reference. If false positive variants were called positions in the core genome between the reference genome and corresponding short reads, these positions were corrected using the consensus sequence provided by the program. The observed variant positions were first checked for support in the variant calling file followed by a repetition of the mapping procedure.

To remove regions of increased SNPs from this alignment, indicative of recombination, the alignment was next processed in *gubbins* v2.4.1 (Croucher et al., 2015) using default parameters, but with maximum 20 iterations of recombination detection. The ML tree was generated by *RAxML* integrated in the *gubbins* based on general time-reversible model of substitution and a gamma distribution to account for rate heterogeneity between sites. SNP positions that were masked by *gubbins* were subsequently excluded by the tool *snp-sites* v2.5.1 (Page et al., 2016). The pairwise SNP distance was determined with *snps-dists* v0.7.0 (Seemann, 2007) based on the

final alignment. The phylogenetic trees were visualized and annotated in *iTOL* (Letunic & Bork, 2019).

Annotation, pangenome analysis, resistance gene detection, alignments

The assemblies were annotated using *PROKKA* v1.14.6 (Seemann, 2014b). The *.gff* files were used for the pangenome determination with *GenAPI* with default settings(Gabrielaite & Marvig, 2020). Gene content analysis was performed in R as described previously by Holt et al. 2015 (Holt et al., 2015).

For the identification of the ESBL-Ec ST131 subclade C1 isolates presence and coverage of the marker, a 1.5 kb M27PP1 prophage-like genomic island, was determined (Birgy et al., 2017). FimH allele typing was done by blasting the draft assemblies against the FimTyper database ((Roer et al., 2017), database downloaded 27.07.2020). AMR conferring genes (ARGs) were identified using *ABRicate* v0.9.9 (Seemann, 2014a) with the integrated Comprehensive Antibiotic Resistance Database (CARD) ((McArthur et al., 2013), database download 20.02.2020). $_{bla}$*CTX-M* genes in assemblies, based on short reads only, were considered to be fragmented if coverage was < 90 %. Isolates were considered to have two $_{bla}$*CTX-M* genes if both were > 90 % coverage.

References

Birgy, Bidet, Levy, Sobral, Cohen, & Bonacorsi. (2017). CTX-M-27–producing escherichia coli of sequence type 131 and clade C1-M27, France. In *Emerging Infectious Diseases* (Vol. 23, Issue 5, p. 885). Centers for Disease Control and Prevention (CDC). https://doi.org/10.3201/eid2305.161865

Croucher, Page, Connor, Delaney, Keane, Bentley, Parkhill, & Harris. (2015). Rapid phylogenetic analysis of large samples of recombinant bacterial whole genome sequences using Gubbins. *Nucleic Acids Research*, *43*(3), e15. https://doi.org/10.1093/nar/gku1196

Gabrielaite, & Marvig. (2020). GenAPI: A tool for gene absence-presence identification in fragmented bacterial genome sequences. *BMC Bioinformatics*, *21*(1), 320. https://doi.org/10.1186/s12859-020-03657-5

Gurevich, A., Saveliev, V., Vyahhi, N., Tesler, G. (2013). QUAST: Quality assessment tool for genome assemblies. *Bioinformatics*, *29*(8), 1072–1075. https://doi.org/10.1093/bioinformatics/btt086

Holt, Wertheim, Zadoks, Baker, Whitehouse, Dance, Jenney, Connor, Hsu, Severin, Brisse, Cao, Wilksch, Gorrie, Schultz, Edwards, Van Nguyen, … Thomson. (2015). Genomic analysis of diversity, population structure, virulence, and antimicrobial resistance in Klebsiella pneumoniae, an urgent threat to public health. *Proceedings of the National Academy of Sciences of the United States of America*, *112*(27), E3574–E3581. https://doi.org/10.1073/pnas.1501049112

Letunic, & Bork. (2019). Interactive Tree of Life (iTOL) v4: Recent updates and new developments. *Nucleic Acids Research*, *47*(W1), 256–259.

https://doi.org/10.1093/nar/gkz239

McArthur, Waglechner, Nizam, Yan, Azad, Baylay, Bhullar, Canova, De Pascale, Ejim, Kalan, King, Koteva, Morar, Mulvey, O’Brien, Pawlowski, … Wright. (2013). The Comprehensive Antibiotic Resistance Database. *Antimicrobial Agents and Chemotherapy*, *57*(7), 3348–3357. https://doi.org/10.1128/AAC.00419-13

Page, Taylor, Delaney, Soares, Seemann, Keane, & Harris. (2016). SNP-sites: rapid efficient extraction of SNPs from multi-FASTA alignments. *Microbial Genomics*, *2*(4), e000056. https://doi.org/10.1099/mgen.0.000056

Roer, Tchesnokova, Allesoe, Muradova, Chattopadhyay, Ahrenfeldt, Thomsen, Lund, Hansen, Hammerum, Sokurenko, & Hasman. (2017). Development of a web tool for Escherichia coli subtyping based on fimh alleles. *Journal of Clinical Microbiology*, *55*(8), 2538–2543. https://doi.org/10.1128/JCM.00737-17

Seemann. (2007). *snp-dists*. https://github.com/tseemann/snp-dists

Seemann. (2014a). *ABRicate*. https://github.com/tseemann/abricate

Seemann. (2014b). Prokka: Rapid prokaryotic genome annotation. *Bioinformatics*, *30*(14), 2068–2069. https://doi.org/10.1093/bioinformatics/btu153

Seemann. (2015). *snippy: fast bacterial variant calling from NGS reads* (4.6.0). https://github.com/tseemann/snippy

Treangen, Ondov, Koren, & Phillippy. (2014). The harvest suite for rapid core-genome alignment and visualization of thousands of intraspecific microbial genomes. *Genome Biology*, *15*(11), 524. https://doi.org/10.1186/s13059-014-0524-x

Figure S1: Core genome phylogeny and genomic features of clinical ESBL-Kp isolates. ML phylogeny of 60 *K. pneumoniae* genomes based on a 4218051 bp core alignment generated with parSNP. The *K. pneumoniae* strains were isolated from overall 49 patients. 41 patients were sampled only once, while 19 isolates were from patients who were sampled multiple times. Isolates (branch tips) are color-coded according to their sequence-type (ST), the presence of different $_{bla}$*CTX-M* alleles and the origin of the sampled material as indicated in the figure key (left, from inner to outer ring). The ST was based on multi-locus sequence typing (MLST) annotation using pubMLST. In addition to the important international outbreak clones of *K. pneumoniae* ST48, ST14 and ST307, we found eleven ESBL-Kp isolates that belonged to ST1626, indicating an important endemic contribution also of non-global ST-types. As observed for the ESBL positive *E. coli* isolates, mostly $_{bla}$*CTX-M*-15 genes (in 57 out of the 60 isolates) were found. Fragments of $_{bla}$*CTX-M-101 and* $_{bla}$*CTX-M-103* genes were detected at a below 90% coverage but not included in the figure. n.t. – not typeable, n.d. – no data available; rare - combined data of several rare ST types with fewer than three members. Three *E. coli* isolates were reclassified as *K. pneumoniae* isolates (rc_ec_xxxxx) based on the WGS data.

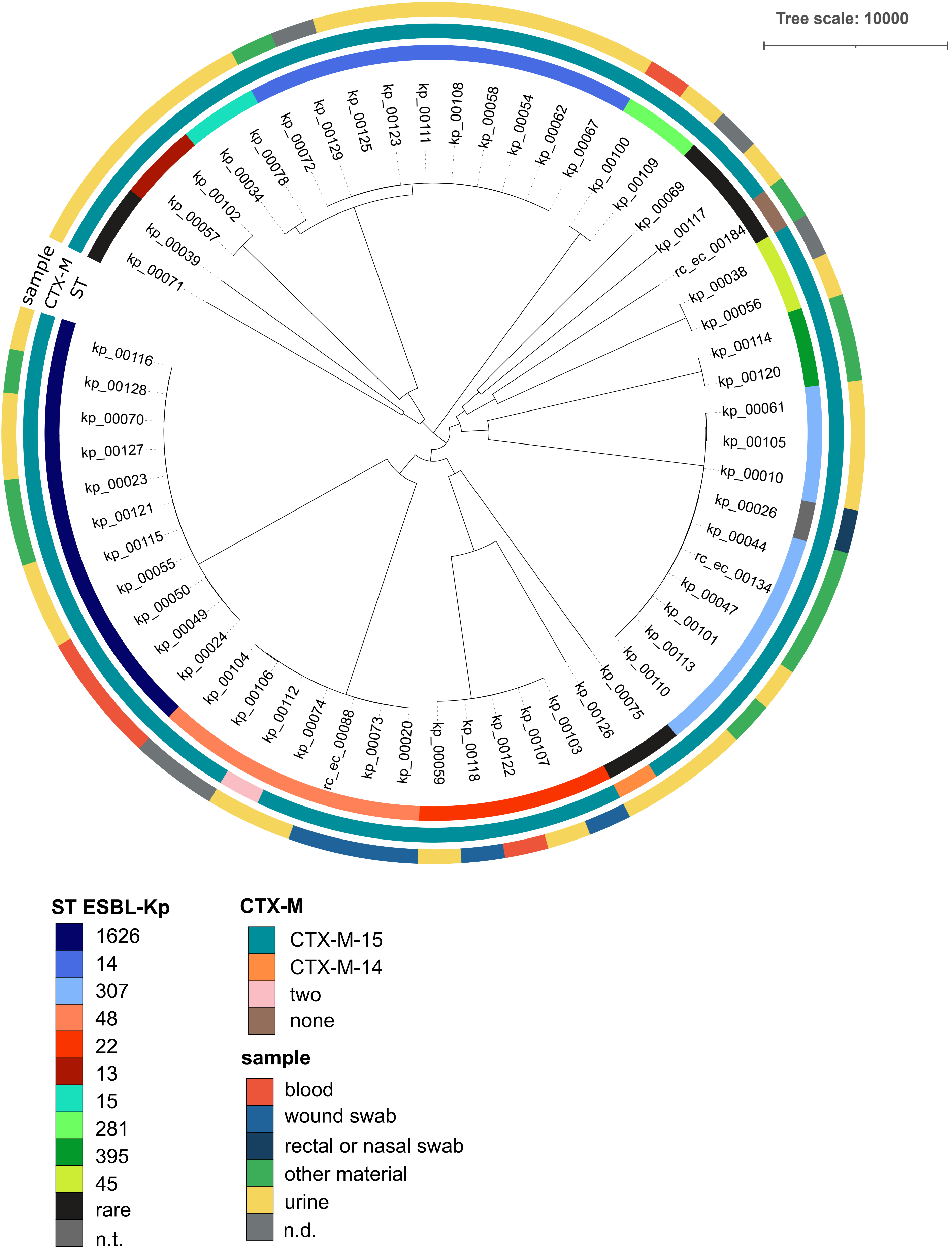

Tree scale: 10000
sample
CTX-M
ST
kp_00071
kp_00039
kp_00057
kp_00102
kp_00034
kp_00078
kp_00072
kp_00129
kp_00125
kp_00123
kp_00111
kp_00108
kp_00058
kp_00054
kp_00062
kp_00067
kp_00100
kp_00109
kp_00069
kp_00117
rc_ec_00184
kp_00038
kp_00056
kp_00114
kp_00120
kp_00061
kp_00105
kp_00010
kp_00026
kp_00044
rc_ec_00134
kp_00047
kp_00101
kp_00113
kp_00110
kp_00075
kp_00126
kp_00103
kp_00107
kp_00122
kp_00118
kp_00059
kp_00020
kp_00073
rc_ec_00088
kp_00074
kp_00112
kp_00106
kp_00104
kp_00024
kp_00049
kp_00050
kp_00055
kp_00115
kp_00121
kp_00023
kp_00127
kp_00070
kp_00128
kp_00116
ST ESBL-Kp
1626
14
307
48
22
13
15
281
395
45
rare
n.t.
CTX-M
CTX-M-15
CTX-M-14
two
none
sample
blood
wound swab
rectal or nasal swab
other material
urine
n.d.

Fig. S2: Pan-genome accumulation curve. 234 clinical ESBL-Ec (red) and 60 ESBL-Kp isolates (blue) were included in the analysis. The ESBL-Ec and ESBL-Kp curves are based on 15414 and 10065 overall gene counts, respectively.

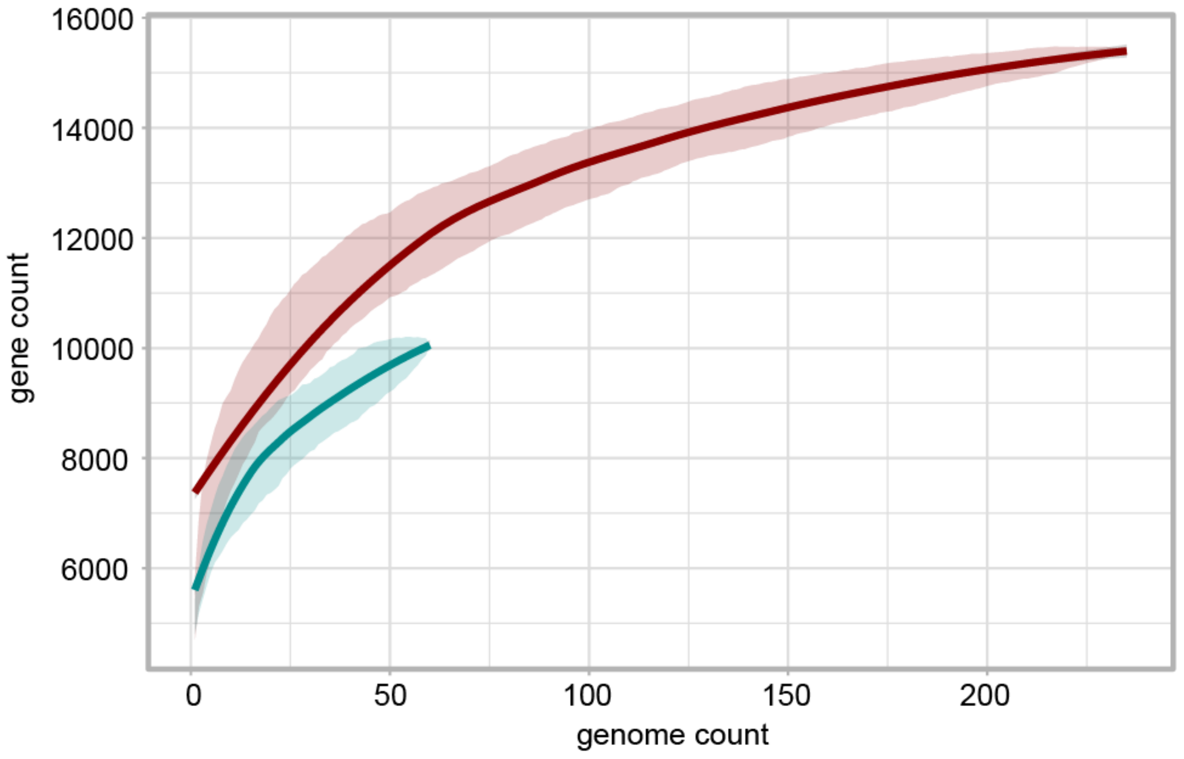

Fig. S3: ST131 ESBL-Ec sub-cluster (SC, n=20) analysis

Maximum likelihood phylogeny of ESBL-Ec genomes of ST 131 built with RAxML 8.2.12. Tree was based on the recombination-free alignment of 3825 bp. Shown here are the 20 SC that contain strains, which have been recovered from different patients.

The *E. coli* isolate ID (first column) as well as the arbitrary patient ID (second column) are given, as well as information on whether the strain was recovered from a nosocomial infection (defined as isolated from the patient > 48 h after admission); nos., dark red: nosocomial, light red: identified within 48h after admission, grey: no information available. w: ward, in which the patient was treated at the time the sample was taken (blue: same ward, white: different ward), h: hospital (yellow: same hospital; red: different hospital). ST131 sub-cluster 1 (SC-1) comprising six strains recovered from three patients that could have had contact.

SNP/Day - Distance Matrix: SNPs (left triangel, blue) and days (right triangle, green) between the isolates

w - ward the patient was treated at the time the sample was taken (blue: same ward, white: different ward)

h - hospital (yellow: central; red: external)

ID - Patient ID, if sampled repeatedly

nos. - isolate appeared non-nosocomial (light orange), nosocomial (orange) or no information available (grey)

clade C1

ST131 SC1	ID	nos.	w	h	SNP/Day	ec_00015	ec_00044	ec_00028	ec_00002	ec_00008	ec_00041
ec_00015	AV				ec_00015		8	10	7	4	9
ec_00044					ec_00044	0		18	15	12	1
ec_00028	AT				ec_00028	1	1		3	6	19
ec_00002					ec_00002	3	3	2		3	16
ec_00008	BB				ec_00008	2	2	1	1		13
ec_00041					ec_00041	2	2	1	1	0	

ST131 SC2	ID	nos.	w	h	SNP/Day	ec_00005	ec_00009	ec_00099
ec_00005	AE				ec_00005		0	38
ec_00009					ec_00009	0		38
ec_00099					ec_00099	0	0	

ST131 SC3	ID	nos.	w	h	SNP/Day	ec_00032	ec_00039
ec_00032			NA		ec_00032		14
ec_00039					ec_00039	0	

ST131 SC4	ID	nos.	w	h	SNP/Day	ec_00150	ec_00208
ec_00150			NA		ec_00150		71
ec_00208					ec_00208	5	

ST131 SC5	ID	nos.	w	h	SNP/Day	ec_00001	ec_00022	ec_00177	ec_00089	ec_00112	ec_00230	ec_00082
ec_00001					ec_00001		2	102	44	102	310	40
ec_00022					ec_00022	2		104	46	104	312	42
ec_00177	BO				ec_00177	8	8		58	0	208	62
ec_00089	AM				ec_00089	8	8	0		58	266	4
ec_00112	BO				ec_00112	8	8	0	0		208	62
ec_00230					ec_00230	7	7	5	5	5		270
ec_00082	AM				ec_00082	10	10	8	8	7	7	

ST131 SC6	ID	nos.	w	h	SNP/Day	ec_00133	ec_00179	ec_00225	ec_00217
ec_00133					ec_00133		56	149	140
ec_00179					ec_00179	0		205	196
ec_00225	BL				ec_00225	2	2		9
ec_00217	BL				ec_00217	2	2	0	

ST131 SC7	ID	nos.	w	h	SNP/Day	ec_00154	ec_00241
ec_00154					ec_00154		152
ec_00241					ec_00241	2	

ST131 SC8	ID	nos.	w	h	SNP/Day	ec_00187	ec_00136	ec_00026	ec_00178
ec_00187					ec_00187		85	255	159
ec_00136					ec_00136	16		170	74
ec_00026					ec_00026	16	28		96
ec_00178					ec_00178	7	19	17	

ST131 SC9	ID	nos.	w	h	SNP/Day	ec_00085	ec_00135	ec_00169
ec_00085					ec_00085		68	110
ec_00135	AZ				ec_00135	5		42
ec_00169	AZ				ec_00169	3	2	

ST131 SC10	ID	nos.	w	h	SNP/Day	ec_00052	ec_00110
ec_00052			NA		ec_00052		21
ec_00110					ec_00110	0	

ST131 SC11	ID	nos.	w	h	SNP/Day	ec_00083	ec_00146	ec_00144	ec_00132	ec_00006	ec_00194	ec_00125
ec_00083					ec_00083		135	138	117	35	222	80
ec_00146					ec_00146	17		3	18	170	87	55
ec_00144	BF				ec_00144	5	12		21	173	84	58
ec_00132	BF				ec_00132	5	12	0		152	105	37
ec_00006					ec_00006	15	22	10	10		257	115
ec_00194	AC				ec_00194	8	15	13	3	13		142
ec_00125	AC				ec_00125	5	12	0	0	10	3	

ST131 SC12	ID	nos.	w	h	SNP/Day	ec_00078	ec_00061	ec_00185
ec_00078					ec_00078		21	200
ec_00061					ec_00061	7		179
ec_00185					ec_00185	3	8	

clade C2

ST131 SC13	ID	nos.	w	h	SNP/Day	ec_00047	ec_00128
ec_00047					ec_00047		104
ec_00128					ec_00128	8	

ST131 SC14	ID		w	h	SNP/Day	ec_00183	ec_00206	ec_00232	ec_00238
ec_00183	AP				ec_00183		1	85	112
ec_00206	AP				ec_00206	15		86	113
ec_00232	AU				ec_00232	15	2		27
ec_00238	AU				ec_00238	17	4	2	

ST131 SC15	ID	nos.	w	h	SNP/Day	ec_00131	ec_00198	ec_00038	ec_00201	ec_00035	ec_00143	ec_00037
ec_00131					ec_00131		107	131	98	123	22	131
ec_00198					ec_00198	3		238	9	230	85	238
ec_00038					ec_00038	3	4		229	8	153	0
ec_00201					ec_00201	7	10	10		221	76	229
ec_00035	BC				ec_00035	7	10	10	6		145	8
ec_00143	AA				ec_00143	13	16	16	12	6		153
ec_00037	BC				ec_00037	13	16	16	12	6	0	

ST131 SC16	ID	nos.	w	h	SNP/Day	ec_00048	ec_00017	ec_00222	ec_00227	ec_00121
ec_00048	AL				ec_00048		4	281	304	127
ec_00017	AL				ec_00017	2		285	308	131
ec_00222	AI				ec_00222	2	4		23	154
ec_00227	AI				ec_00227	2	4	0		177
ec_00121					ec_00121	1	3	3	3	

ST131 SC17	ID	nos.	w	h	SNP/Day	ec_00193	ec_00147	ec_00159	ec_00160	ec_00162	ec_00149
ec_00193					ec_00193		90	63	63	62	64
ec_00147	AQ				ec_00147	5		27	27	28	26
ec_00159	AQ				ec_00159	5	0		0	1	1
ec_00160					ec_00160	5	0	0		1	1
ec_00162	AQ				ec_00162	5	0	0	0		2
ec_00149	AQ				ec_00149	5	0	0	0	0	

ST131 SC18	ID	nos.	w	h	SNP/Day	ec_00010	ec_00246
ec_00010					ec_00010		331
ec_00246					ec_00246	10	

ST131 SC19	ID	nos.	w	h	SNP/Day	ec_00107	ec_00255
ec_00107			NA		ec_00107		256
ec_00255					ec_00255	5	

clade A

ST131 SC20	ID	nos.	w	h	SNP/Day	ec_00216	ec_00226
ec_00216					ec_00216		10
ec_00226					ec_00226	2	

Fig. S4: Non-ST131 ESBL-Ec sub-cluster analysis

Maximum likelihood phylogeny of ESBL-Ec genomes of indicated STs built with RAxML 8.2.12. Midpoint rooted trees were based on the recombination-free alignment of 310 bp (ST1193), 12438 bp (ST88), 3288 bp (ST410), 12329 bp (ST648), 10744 bp (ST69) and 37233 bp (ST10 clonal complex) respectively.

Sub-clusters of isolates (isolate ID on the branch tips) were identified and featured by red lines if they differed by fewer than 10 SNPs. Arbitrary two-letter codes were assigned to individual patients so that isolates that were recovered from the same patients can be followed. Nosocomial (nos.): isolates were recovered from patients > 48 h after admission (light orange). Non-nosocomial (non-nos.): isolates that were recovered from patients within the first 48 h after admission (orange).

SNP/Day - Distance Matrix: SNPs (left triangel, blue) and days (right triangle, green) between the isolates
w - ward the patient was treated at the time the sample was taken (blue: same ward, white: different ward)
h - hospital (yellow: central; red: external)
ID - Patient ID, if sampled repeatedly

nos. - isolate appeared non-nosocomial (light orange), nosocomial (orange) or no information available (grey)

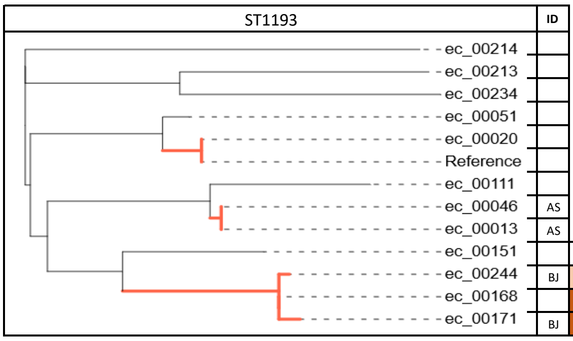

nos.	w	h	SNP/Day	ec_00244	ec_00168	ec_00171
			ec_00244		133	122
			ec_00168	2		21
			ec_00171	6	4	

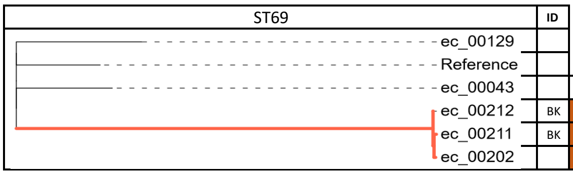

nos.	w	h	SNP/Day	ec_00212	ec_00211	ec_00202
			ec_00212		1	32
			ec_00211	2		31
			ec_00202	2	2	

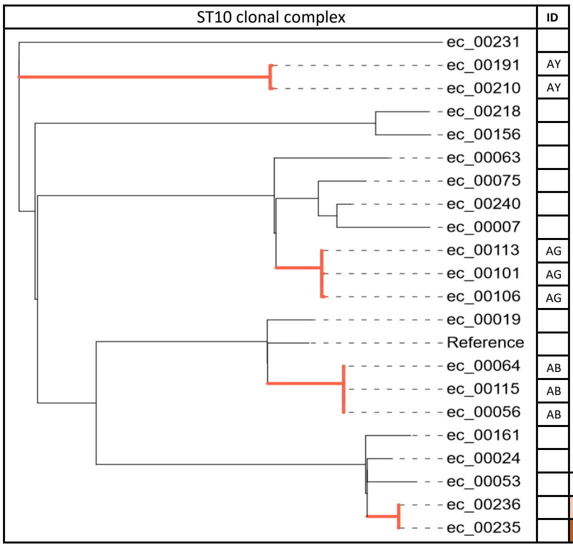

nos.	w	h	SNP/Day	ec_00235	ec_00236
			ec_00235		1
			ec_00236	0	

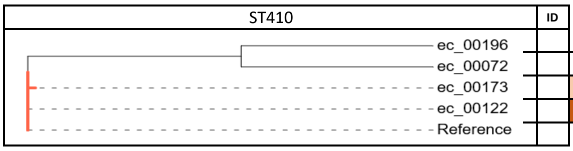

nos.	w	h	SNP/Day	ec_00173	ec_00122
			ec_00173		91
			ec_00122	7	

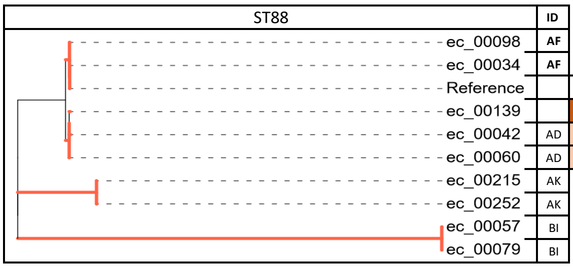

nos.	w	h	SNP/Day	ec_00139	ec_00042	ec_00060
			ec_00139		140	78
			ec_00042	13		62
			ec_00060	6	9	

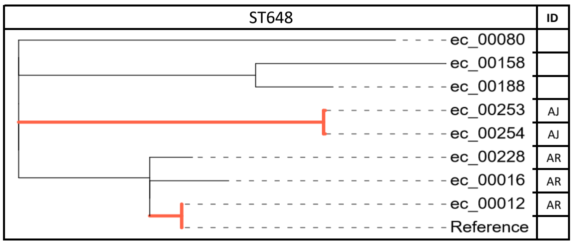

Fig. S5: ESBL-Kp sub-cluster analysis

Maximum likelihood phylogeny of ESBL-Ec genomes of indicated STs built with RAxML 8.2.12. Midpoint rooted trees were based on the recombination-free alignment of 49 bp (ST14), 12 bp (ST22), 145 bp (ST48), and 892 bp (ST307) respectively.

Sub-clusters of isolates (isolate ID on the branch tips) were identified and featured by red lines if they differed by fewer than 10 SNPs. Arbitrary two-letter codes were assigned to individual patients so that isolates that were recovered from the same patients can be followed. Nosocomial (nos.): isolates were recovered from patients > 48 h after admission (light orange). Non-nosocomial (non-nos.): isolates that were recovered from patients within the first 48 h after admission (orange).

SNP/Day - Distance Matrix: SNPs (left triangel, blue) and days (right triangle, green) between the isolates
w - ward the patient was treated at the time the sample was taken (blue: same ward, white: different ward)
h - hospital (yellow: central; red: external)
ID - Patient ID, if sampled repeatedly
nos. - isolate brought to the hospital (light orange), appeared nosocomial (orange), no information available (grey)
*** patients had contact prior to isolate collection**

ST1626

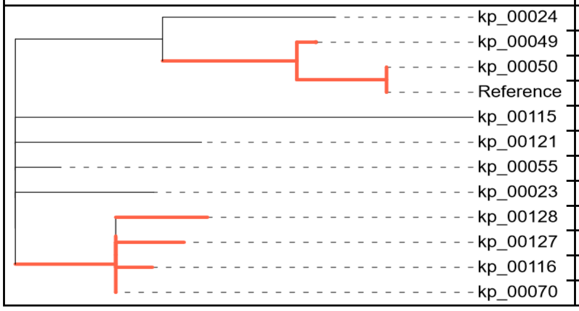

Isolate	ID
kp_00024	
kp_00049	AD
kp_00050	AD
Reference	
kp_00115	
kp_00121	
kp_00055	
kp_00023	
kp_00128	
kp_00127	
kp_00116	
kp_00070	

nos.	w	h	SNP/Day	kp_00128	kp_00127	kp_00116	kp_00070
			kp_00128		11	60	162
			kp_00127	13		71	173
			kp_00116	11	8		102
			kp_00070	8	5	3	

ST22

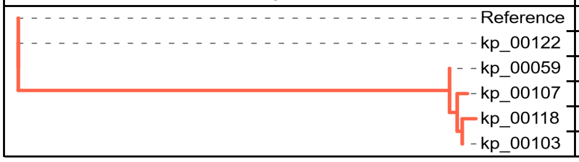

Isolate	ID
Reference	
kp_00122	
kp_00059	AH
kp_00107	AH
kp_00118	AH
kp_00103	

nos.	w	h	SNP/Day	kp_00122	kp_00059	kp_00107	kp_00118	kp_00103
	*		kp_00122		217	109	60	124
	*		kp_00059	8		108	157	93
			kp_00107	8	2		49	15
			kp_00118	10	4	4		64
			kp_00103	8	2	2	2	

ST48

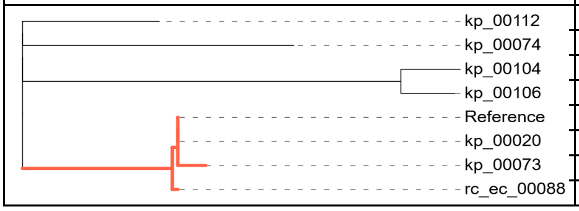

Isolate	ID
kp_00112	
kp_00074	
kp_00104	
kp_00106	
Reference	
kp_00020	BG
kp_00073	
rc_ec_00088	BG

nos.	w	h	SNP/Day	kp_00020	kp_00073	rc_00088
	*		kp_00020		148	0
	*		kp_00073	5		148
			rc_00088	1	6	

ST307

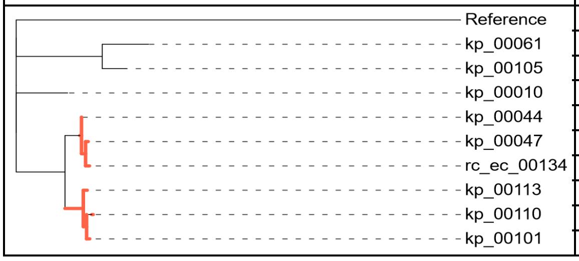

Isolate	ID
Reference	
kp_00061	
kp_00105	
kp_00010	
kp_00044	AB
kp_00047	AB
rc_ec_00134	AB
kp_00113	
kp_00110	AC
kp_00101	AC

nos.	w	h	SNP/Day	kp_00044	kp_00047	rc_00134	kp_00113	kp_00110	kp_00101
			kp_00044		4	4	184	178	151
			kp_00047	3		0	180	174	147
			rc_00134	3	0		180	174	147
			kp_00113	20	23	23		6	33
			kp_00110	22	25	25	8		27
			kp_00101	19	22	22	5	5	

Fig. S6: SNP differences between ESBL-Ec samples from the same patient. Arbitrary two letter codes were assigned to those patients for whom at least two samples of the same ST were included in this study (colors reflect the various ST). Numbers in white spaces between the *E. coli* isolate identification (ID) numbers are pairwise SNP distances; if no space is between the *E. coli* IDs, the SNP distance is zero.

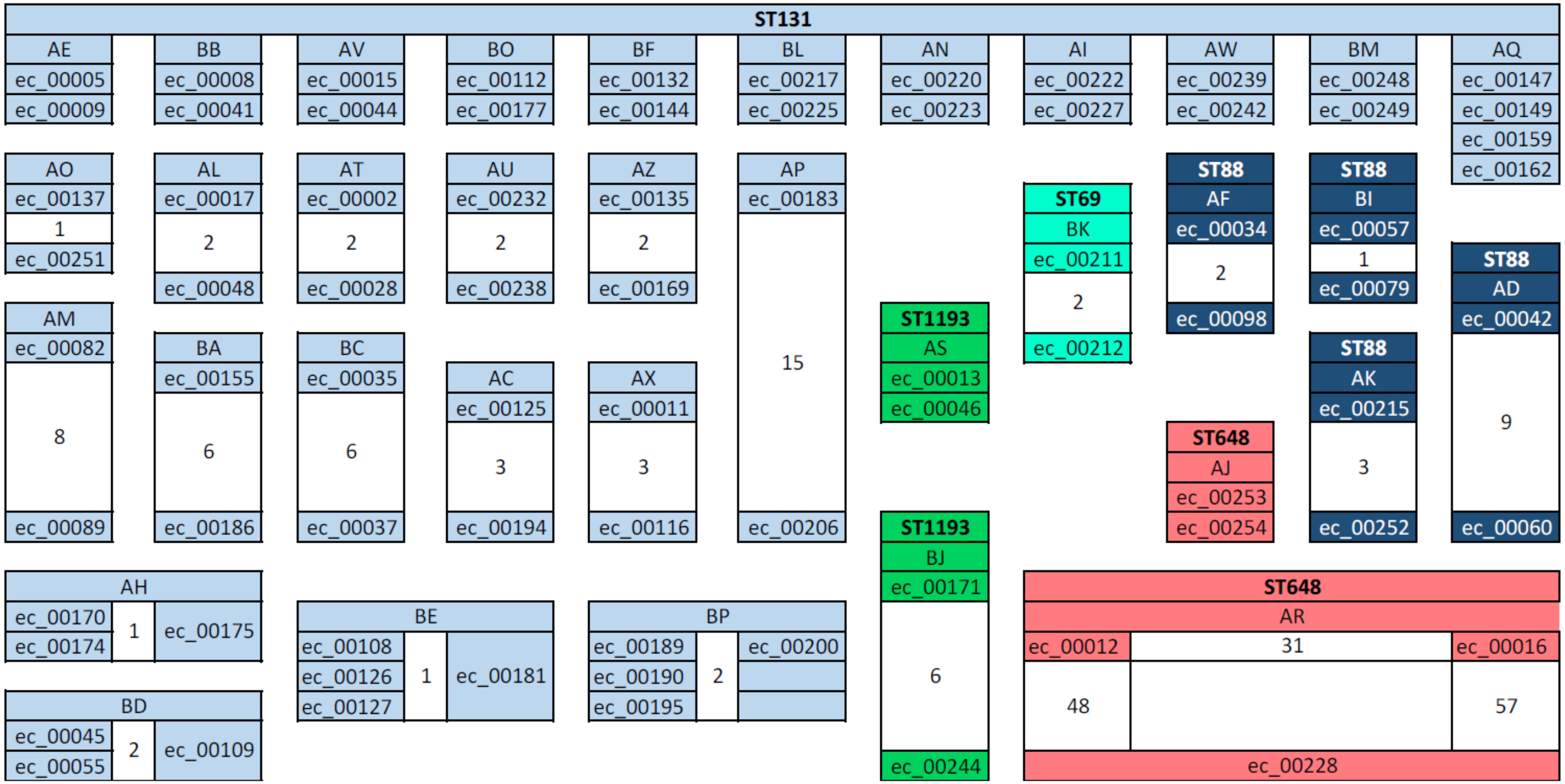

Tab. S1: Selection of sequence-type (ST)-specific *E. coli* (Ec) and *K. pneumoniae* (Kp) reference genomes used for short-read mapping of the other clinical isolates of the respective ST analyzed in this study. These were generated in this study through hybrid Illumina/MinION assembly, unless otherwise indicated (external). The numbers of clinical isolates belonging to the various sequence types as well as the maximum SNP difference between any two clinical isolates within the respective ST-group are given. The proportion of the core positions that were found in all isolates of the respective ST as compared to the overall positions of the chosen reference genome is also presented.

Species	ST	Reference genome	Number of isolates	Max SNP dist.	core positions
Ec	131	ec_00010	144	362	91 %
Ec	1193	ec_00020	12	112	98 %
Ec	88	ec_00034	9	1030	89 %
Ec	410	ec_00122	4	139	92 %
Ec	ST10 cc.	External (accession: GCA_000814145.2)	21	434	89 %
Ec	69	External (accession: GCA_902668645)	5	289	89 %
Ec	648	ec_00012	8	268	91 %
Kp	1626	kp_00050	11	53	99 %
Kp	307	External (accession: CP046612)	9	230	92 %
Kp	22	kp_00122	5	12	98 %
Kp	48	kp_00020	7	87	97 %

Tab. S2: Information on the clinical isolates. A, C: Patient ID (Pat. ID.) are arbitrarily given letters for patients that were sampled repeatedly. ST was determined *in silico* using pubMLST. Date of isolation (year-month-day) and carriage status of the isolate as determined by the hospital are given**. B, D**: Assembly quality statistics of the isolate collection.
